# Genetic evidence that the Makira region in northeastern Madagascar is a hotspot of malaria transmission

**DOI:** 10.1186/s12936-016-1644-4

**Published:** 2016-12-20

**Authors:** Benjamin L. Rice, Christopher D. Golden, Evelin Jean Gasta Anjaranirina, Carolina Mastella Botelho, Sarah K. Volkman, Daniel L. Hartl

**Affiliations:** 1 Department of Organismic and Evolutionary Biology, Harvard University, Cambridge, MA USA; 2 Department of Environmental Health, Harvard T.H. Chan School of Public Health, Boston, MA USA; 3 Harvard University Center for the Environment, Cambridge, MA USA; 4 Madagascar Health and Environmental Research (MAHERY), Maroantsetra, Madagascar; 5 Department of Pharmacy and Nutrition, Federal University of Espirito Santo, Alegre, ES Brazil; 6 Department of Immunology and Infectious Diseases, Harvard T.H. Chan School of Public Health, Boston, MA USA

**Keywords:** Madagascar, *Plasmodium falciparum*, Genetic diversity, Genetic surveillance, Polygenomic infections

## Abstract

**Background:**

Encouraging advances in the control of *Plasmodium falciparum* malaria have been observed across much of Africa in the past decade. However, regions of high relative prevalence and transmission that remain unaddressed or unrecognized provide a threat to this progress. Difficulties in identifying such localized hotspots include inadequate surveillance, especially in remote regions, and the cost and labor needed to produce direct estimates of transmission. Genetic data can provide a much-needed alternative to such empirical estimates, as the pattern of genetic variation within malaria parasite populations is indicative of the level of local transmission. Here, genetic data were used to provide the first empirical estimates of *P. falciparum* malaria prevalence and transmission dynamics for the rural, remote Makira region of northeastern Madagascar.

**Methods:**

Longitudinal surveys of a cohort of 698 total individuals (both sexes, 0–74 years of age) were performed in two communities bordering the Makira Natural Park protected area. Rapid diagnostic tests, with confirmation by molecular methods, were used to estimate *P. falciparum* prevalence at three seasonal time points separated by 4-month intervals. Genomic loci in a panel of polymorphic, putatively neutral markers were genotyped for 94 *P. falciparum* infections and used to characterize genetic parameters known to correlate with transmission levels.

**Results:**

Overall, 27.8% of individuals tested positive for *P. falciparum* over the 10-month course of the study, a rate approximately sevenfold higher than the countrywide average for Madagascar. Among those *P. falciparum* infections, a high level of genotypic diversity and a high frequency of polygenomic infections (68.1%) were observed, providing a pattern consistent with high and stable transmission.

**Conclusions:**

Prevalence and genetic diversity data indicate that the Makira region is a hotspot of *P. falciparum* transmission in Madagascar. This suggests that the area should be highlighted for future interventions and that additional areas of high transmission may be present in ecologically similar regions nearby.

**Electronic supplementary material:**

The online version of this article (doi:10.1186/s12936-016-1644-4) contains supplementary material, which is available to authorized users.

## Background

Approximately 300,000 cases of *Plasmodium falciparum* malaria are recorded annually in Madagascar, an incidence rate that, at the reported level, places Madagascar and its population of 24.9 million among the lower tier of African countries in terms of malaria burden [1]. Updated estimates of transmission across Africa based on large databases of field surveys and sophisticated model-based geostatistics are now available and are less dependent on clinical reporting. These model-based estimates indicate that the number of reported cases in Madagascar is likely a large underestimate of the true burden, but still rank Madagascar as a low transmission setting relative to much of mainland Africa [2].

Contrasting with these low estimates for Madagascar are recent longitudinal and cross-sectional cohort studies that have repeatedly found localities with transmission and prevalence rates 3- to 10-fold higher than the national average (for example, see [3–5]). Further, these estimates are comparable with recognized high transmission areas globally. The localities of higher transmission and prevalence observed in Madagascar were often found in rural areas in the eastern part of the country, which is primarily characterized by lowland tropical rainforest. For example, prevalence of *P. falciparum* was 20.5% in a cohort of pregnant women reporting to antenatal clinics near Manakara [5] and varied temporally from 19.7 to 35.2% among adults in the Alaotra-Mangoro region [6]. For comparison, the average prevalence estimated from monitoring sentinel health sites was 3.1% nationally and peaked at 4.9% in the eastern part of the country [7]. Additionally, transmission was found to be newly increasing in some areas of eastern Madagascar, with a large outbreak in the Vatovavy-Fitovinany and Atsimo-Atsinanana regions in 2011–2012 as an example [8].

In terms of malaria transmission, Madagascar is typically divided into four to five zones based on broad climatic and demographic regions: the south, the eastern coast, the western coast, the high central plateau and sometimes with the margins/periphery of the high plateau included as a separate zone [7, 9, 10]. Transmission rates have been found to be highest along the eastern coast (4.9–11.7%) or the western coast (4.6–14.3%), with estimates varying with the age group and seasonality of sampling [7, 9].

The continued reports of localized areas of elevated transmission, sometimes termed foci [11, 12] or hotspots [13], which far exceed those national or regional level estimates suggest that monitoring efforts, and progress in reducing the burden of malaria, have been unevenly distributed. If unaddressed, such hotspots have the potential to undermine the effect of the recent scaling-up of malaria control in Madagascar [13, 14]. Given the historical context of low intervention coverage and low funding for interventions in Madagascar in comparison to other African countries [1, 15, 16], identifying such hotspots of transmission is also valuable in order to efficiently direct resources to the areas where scale-up is most urgently needed. However, to do this, a better understanding of both the geographic distribution and transmission dynamics of such hotspots within Madagascar is required.

In terms of geographic distribution, a majority of the cohort studies that have measured malaria transmission and prevalence were performed in the southern parts of eastern Madagascar and, although the areas are climatically similar, it is unknown if comparable results are to be expected in the Northeast. Additionally, much of the population of the Northeast lives in rural and remote forested districts that are not well monitored by national surveillance efforts [3, 9].

In terms of transmission dynamics, it is also unknown if local hotspots within Madagascar are the result of short-term spikes in transmission triggered by transient changes in ecological or epidemiological conditions, or are due temporally stable transmission. Genetic data can be of great utility in discriminating between the two transmission scenarios without the need for labor-intensive direct estimates of transmission. The discrimination is possible because different levels of transmission intensity and stability are expected to produce different patterns of genetic variation in the *Plasmodium* parasite population [17–19]. Specifically, high, stable transmission is expected to result in a diverse assemblage of parasite genomes circulating locally, and a high percentage of infections that contain multiple, distinct *P. falciparum* genomes concurrently (termed polygenomic infections). On the other hand, in situations with low or sporadic transmission, few hosts on average are expected to encounter multiple, distinct *P. falciparum* multi-locus genotypes (MLGs) during the course of an infection, reducing the proportion of infections that are polygenomic. As a result, when transmission is unstable, opportunities for re-assortment and recombination between parasite MLGs are rare and few distinct MLGs circulate. Sporadic outbreaks, although capable of infecting a large percentage of the population when they do occur, are then expected to be composed of relatively few MLGs (albeit with some at potentially high frequency).

Here, the observation of an apparent hotspot of malaria prevalence in northeastern Madagascar is reported and a pattern of genetic variation consistent with high, stable transmission observed. This is inferred from rapid diagnostic test (RDT) surveys and single nucleotide polymorphism (SNP) genotyping of 94 *P. falciparum* positive samples from a prospective cohort study of 698 individuals living in the remote Makira region. These data highlight the need for greater monitoring of a previously understudied area in Madagascar and provide a baseline against which future interventions can be measured.

## Methods

### Study sites and study population

**Fig. 1:**
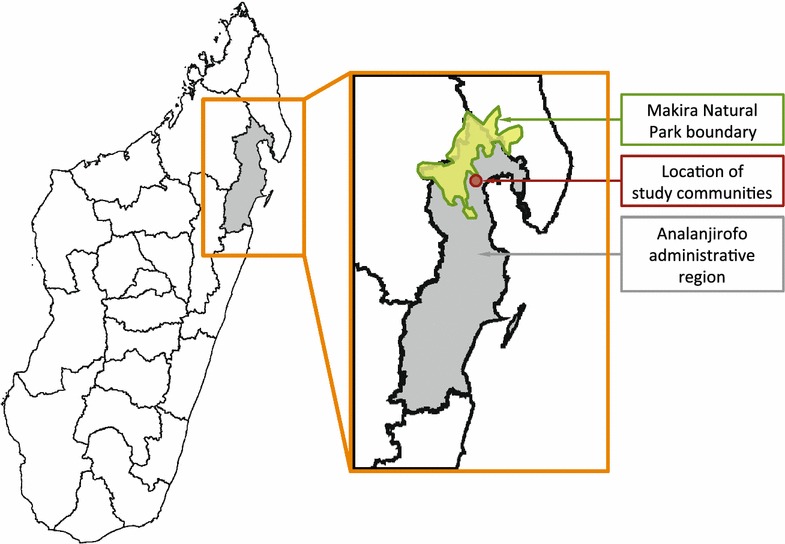
Location of the Makira Region and the study communities in northeastern Madagascar. The Analanjirofo administrative region (*faritra*) is colored *gray*. The approximate boundary of the Makira Natural Park is shown in *green*. Approximate location of the study communities is shown in *red *

The Makira region is a large rural area mostly within the Analanjirofo administrative region (*faritra*) in northeastern Madagascar. It contains the protected Makira Natural Park (372,470 ha) and buffer zones (350,000 ha) in the surrounding low- and medium-altitude forest [20]. The park and buffer zones combined have an area of approximately 722,000 hectares and support a population of more than 127,000 people [21]. This area has been previously studied as a part of an effort to understand linkages between the conservation of its extraordinary biodiversity and human health (for e.g., see [20, 22]). Communities in the Makira region consist of small, dense clusters of approximately 50–200 households. Two communities (*fokontany*) were selected. For the privacy of study participants, the names of these communities are withheld per the recommendations of the ethical body that reviewed the study and in accordance with previous studies that have been performed in these communities [22]. See Fig. 1 for the approximate geographic locations. The two communities are separated by 7.2 km; however, community 2 is at a notably higher elevation (350 m) than community 1 (20 m). Neither community has road access and the distance to the nearest reporting sentinel health site (see Ref. [7]) is 23–32 km. Households were randomly selected within the communities. All individuals in 97 and 57 households, in community 1 and community 2, respectively, were enrolled for a total of 698 individuals.

Ethical approval was given by the Madagascar Ministry of Health and the Office for the Protection of Human Subjects at the Harvard T.H. Chan School of Public Health (IRB 22826). Approval for the study was also sought and obtained from the Maroantsetra regional medical inspector, the mayor covering the region including both communities, and the leader (president-fokontany) in each community. All adults provided informed consent for themselves and surrogate consent for infants and children, while other older children provided assent prior to interaction with the local research staff.

### Sample collection

For each individual, a blood sample was obtained by venous blood draw, or by finger-prick or heel-prick in a few cases for younger children. Five microlitres of blood were added to an RDT and approximately 200 ml of blood were added to an FTA card (Whatman FTA Classic, GE Healthcare, Marlborough, MA, USA, cat. no. WB120312) to preserve DNA for later genetic analyses. The RDTs used were: First Response® Combo Pf/Pan (Premier Medical Corporation Limited, Kachigam, UT, India, cat. no. I 16 FRC), CareStart® Pf/Pan (ACCESS BIO, Inc., Somerset, NJ, USA, cat. no. G0131), and SD BIOLINE® Pf/Pan (Standard Diagnostics, Gyeonggi-do, Korea, cat. no. 05FK60)—all of which used the antigens HRP2 and pLDH. All individuals testing positive by RDT were offered treatment with the standard first-line treatment (artesunate + amodiaquine, AS + AQ). Blood spots on FTA cards were allowed to dry and then stored at ambient temperature with desiccant in plastic bags.

Individuals were sampled at up to three time points: time point 1 (July/August 2013), time point 2 (November/December 2013), and time point 3 (March/April 2014). These sampling times correspond to the medium, low and high seasons of rainfall in the area, respectively. The number of individuals with a valid RDT result varied from 495 to 660 per time point; 698 individuals had an RDT result for at least one time point and 408 had an RDT result for all three time points.

### DNA extraction and molecular detection of *Plasmodium* species

For a randomly chosen subset (*n* = 166) of the samples positive by RDT for *P. falciparum*, DNA was extracted from the corresponding dried blood spots using the DNA IQ™ Casework Pro for Maxwell® 16 kit. Following DNA extraction, real-time PCR (RT-PCR) assays for *P. falciparum, Plasmodium vivax, Plasmodium malariae,* and *Plasmodium ovale* were performed in order to confirm the presence of *Plasmodium* parasite genetic material. The assay for *P. falciparum* was as described previously [23], while the assays for the other three species followed a similar protocol targeting the conserved plasmepsin gene [24]. *Plasmodium falciparum, P. vivax, P. malariae,* and *P. ovale* are known to be distributed throughout Madagascar, although *P. falciparum* is estimated to account for 96% of cases [1]. Assessing the agreement between RDT results and molecular detection of *Plasmodium* species is warranted, as the specificity of RDTs is known to be variable, especially if multiple *Plasmodium* species are present [25–28].

### Genetic sampling: SNP-genotyping analysis

#### Calling alleles

For the confirmed *P. falciparum* infections, alleles from a panel of 24 putatively neutral and unlinked genomic loci were typed to produce multi-locus genotypes (MLGs) [23]. Twelve or more loci were typed for 94 samples (see “Results” section). These loci and the SNP-genotyping protocol used have been termed a *P. falciparum* “molecular barcode” and are described elsewhere [18, 23, 29, 30]. The loci are distributed on 12 of the 14 *P. falciparum* chromosomes (see Additional file 1: Table S1) and are identical to those of Daniels et al. [23]. Briefly, the presence of a variant allele at the targeted locus causes a difference in the melting temperature of an RT-PCR probe that can be detected using high-resolution melting (HRM) analysis. If, for a sample, two alleles are present at the probed locus, two melting peaks, corresponding to the two alleles present, are observed. As *Plasmodium* parasites are haploid in the bloodstream stage of their life cycle, observing two alleles for a locus indicates that multiple distinct parasite genomes were present in the host at the time of sampling. Such infections are reported as polygenomic infections. To be conservative, and consistent with previous conventions, an infection is reported as polygenomic if at least two of the loci analyzed for that sample exhibit the two melting peaks indicative of two alleles present [18, 23].

As the concentration of parasite DNA in blood samples can be quite low relative to other co-infecting parasites, or relative to host DNA, a pre-amplification step was performed prior to SNP-genotyping. Pre-amplification was previously shown to increase the success rate and sensitivity of SNP-genotyping (method same as used in Ref. [31]).

### Genetic correlates of transmission

Two genetic parameters hypothesized to reflect transmission dynamics and empirically shown to co-vary with transmission intensity are (1) the percentage of infections that are polygenomic and (2) the frequency distribution of MLGs [18, 19]. When transmission is low or unstable, fewer distinct MLGs are present locally and more individuals contain a MLG identical to one that has already been sampled. This leads to some MLGs being observed at elevated frequencies within the sample. In contrast, at higher and more stable transmission levels, few infections contain repeatedly observed MLGs.

### Estimating minor allele frequencies and resolution

In order to evaluate the resolution of our genetic sampling, we first estimated the minor allele frequency (MAF) at each of the 24 loci. To account for the fact that polygenomic samples were present and that the multiplicity of infection within polygenomic samples was unknown, a form of the “predominant allele” approach was used [32]. For polygenomic samples, those loci where multiple alleles were present were not considered, but the loci where only one allele was observed (indicating that the MLGs forming the polygenomic infection had the same allele at the locus) were considered. Tallying predominant alleles has the advantage of allowing the alleles in polygenomic infection samples (which can be a large proportion of the sample) to be taken into consideration when determining MAF. This approach has also been empirically shown to correlate closely with other measures of allele frequency [32, 33], but does include an assumption that allele frequencies at the observed “monogenomic” loci are not strongly biased.

Assuming alleles at a locus are neutral and segregate independently, the probability that two unrelated parasite lineages would be observed to have the same allele by chance is given by MAF^2^ + (1 − MAF)^2^. For multiple loci, the probability of observing an identical MLG by chance (identity by state, IBS) is simply the product of IBS probabilities for each locus. As the average MAF for the loci analysed approaches its maximum of 0.5 (indicating 50% of the samples have the alternate allele), the number of loci required for a significant level of resolution decreases. Using a significance cut-off of α = 0.01 and the derived estimate of the MAF for each locus, the number of loci needed to have a significantly small probability (i.e., <0.01) of randomly observing identical MLGs was determined if: (1) the loci with the highest observed MAFs were used, or (2) the loci with the lowest observed MAFs were used. For (1) and (2), these represent the fewest and the most loci needed, respectively, to achieve the chosen level of confidence given the observed MAFs.

### Estimating multi-locus genotype frequencies and genetic similarity

To identify any samples with identical MLGs and to estimate the genetic similarity among samples, pairwise percent identity was calculated (using a Python script, available upon request) for all samples and for subsets of samples of interest. For polygenomic infections, we calculated genetic similarity among the *P. falciparum* MLGs within the infection as the proportion of typed loci for that infection that shared the same allele. To account for differences in the number of pairwise comparisons used to calculate mean genetic similarity for different subsets of samples, bootstrap resampling was used to test for significance with subsamples equal in size to the number of comparisons made in the smallest subset (in R, script available upon request). *P* values are reported as the proportion of subsamples of a given size that had a mean within the standard error of the mean of the other subset of samples.

## Results

### High prevalence of *P. falciparum* infection by RDT

**Fig. 2:**
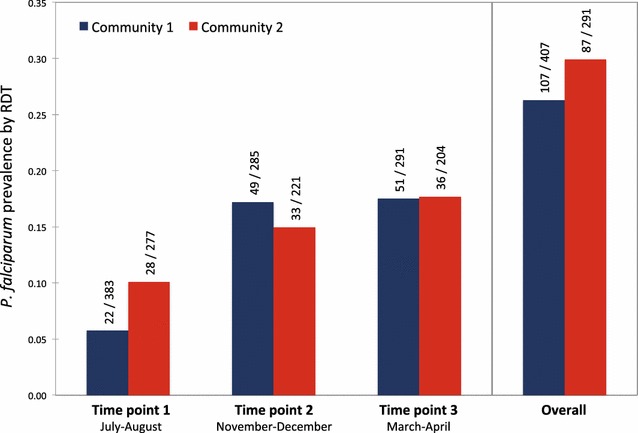
High relative prevalence and temporal variation of *P. falciparum* in the Makira region of Madagascar. Prevalence of *P. falciparum* by RDT is shown with community 1 in *blue* and community 2 in *red*. The number of positive samples out of the number of individuals surveyed is shown above. Shown to the *right* is the overall proportion of individuals that tested positive at one or more time point during the course of the study. The overall percentage of individuals positive for both communities combined was 27.8%

For both communities, prevalence by RDT varied temporally, from a low of 5.7–10.1% in July–August to a high of 17–18% in March–April for each community (see Fig. 2). Overall, 26.3 and 29.9% of individuals for community 1 and community 2, respectively, tested positive for *P. falciparum* by RDT during the course of the 10-month follow-up. For both communities combined, there were 504 individuals (72.2%) with a negative *P. falciparum* RDT result at all three time points. Among individuals with a positive RDT result, 169 (24.2%), 20 (2.9%), 4 (0.6%), and 1 (0.1%) individuals were positive at one time point, two consecutive time points, two nonconsecutive time points, and all three time points, respectively. Among children under five, 34 of 134 children sampled (25.4%) were RDT positive. At the household level, 63.0% of households had at least one member of the household test positive for *P. falciparum* by RDT.

For the randomly chosen subset (*n* = 166) of the samples positive by RDT for *P. falciparum* that were analysed, the presence of *P. falciparum* was identified by RT-PCR in 88% of the samples. This supports general agreement between RDT and RT-PCR results and is comparable to other analyses of RT-PCR and RDT concordance in Madagascar (e.g. 79.5% in the national 2013 Malaria Indicator Survey [9]). Possible explanations for the 12% of RDT positive samples that were not positive for *P. falciparum* by RT-PCR include: (1) false positive RDTs, (2) a failure to preserve DNA between the time of sampling and the later RT-PCR analysis for some samples, (3) failed RT-PCR reactions and (4) infections due to other *Plasmodium* species. Apart from *P. falciparum*, two samples were positive for *P. vivax* by RT-PCR, one from each community at the third time point. The *P. vivax* positive sample from community 1 was also positive for *P. falciparum* and thus a mixed-species infection. Furthermore, two samples from community 1 were positive for *P. malariae*. The *P. malariae* sample from time point 1 was found to also harbour a polygenomic infection of *P. falciparum*. This sample was therefore both a mixed-species infection as well as a complex *P. falciparum* infection (see more discussion of polygenomic *P. falciparum* infections below).

### Frequency of polygenomic infections

In addition to observing high prevalence, we also observed that a high frequency of infections were polygenomic (68.1%) among the 94 *P. falciparum* infections that were typed at 12 or more loci (see Table 1; Additional file 2). This observation was replicated across communities, across time points, across sexes, and across subjects’ age groups (range 59.6–79.5%). The percentage of infections found to be polygenomic was also similar among the samples typed at 12–17 loci (68.5%) and the samples that were typed at 18 or more loci (66.7%). The number of SNP positions that were polymorphic within infections (i.e. polygenomic) ranged from 0 to 11. The mean and median number of polymorphic loci among polygenomic samples was 3.95 and 3, respectively.

**Table 1:** Frequency of polygenomic *Plasmodium falciparum* infections in the Makira region of Madagascar

Category	Group	*n* ^a^	% Polygenomic
Overall	All samples	94	68.1
Community	Community 1	60	61.7
	Community 2	34	79.4
Time point	Time point 1	29	65.5
	Time point 2	43	72.1
	Time point 3	22	63.6
Sex^b^	M	41	78.0
	F	52	59.6
Age^b^	2–12 years	27	70.4
	13+ years	66	66.7
Genetic sampling	12–17 loci typed	73	68.5
	18+ loci typed	21	66.7

^a^ *n* is the number of infections genotyped

^b^Age and sex data was not recorded for one individual

Although the frequency of polygenomic infections was higher in community 2 (79.4%) than in community 1 (61.7%), this difference was not statistically significant (*p* = 0.612, Fisher exact test for count data). Neither was the difference between polygenomic infection frequency among males (78.0%) and females (59.6%) (*p* = 0.075) or that between any pair of time points (*p* > 0.05). The failure to observe a significant difference between time points is notable, as rainfall differs fourfold between time points 2 and 3 and prevalence was observed to change approximately threefold between time points 1 and 3. These data indicate that some factor other than precipitation drives temporal changes in prevalence and that the frequency of polygenomic infections may be seasonally stable.

### SNP allele frequency and validation

In order to permit downstream genetic analysis, it was first validated that the loci assayed were polymorphic. Then the number of loci needed for a given level of resolution in distinguishing parasite MLGs was estimated. All 24 loci analysed were found to be polymorphic, with a high MAF on average (mean MAF 0.310, range 0.083–0.500). The alleles and allele frequencies for each locus are shown in Additional file 1: Table S1. The mean number of loci typed per sample was 15.6, with a range from 12 to 22 loci (see below for the use of 12 loci as the minimum cut-off). For no sample were all 24 loci typed, but MAF was calculated from a mean of 61.3 samples per locus for all 24 loci (range 15–87). Loci A6 and A10, with alleles being callable from only 15 and 16 samples each, respectively, had significantly lower reporting rates than the other loci. Inefficient binding of primers or probes for these loci, possibly due to a variant sequence circulating in the population, may explain the lower number of samples successfully typed for these loci.

**Fig. 3:**
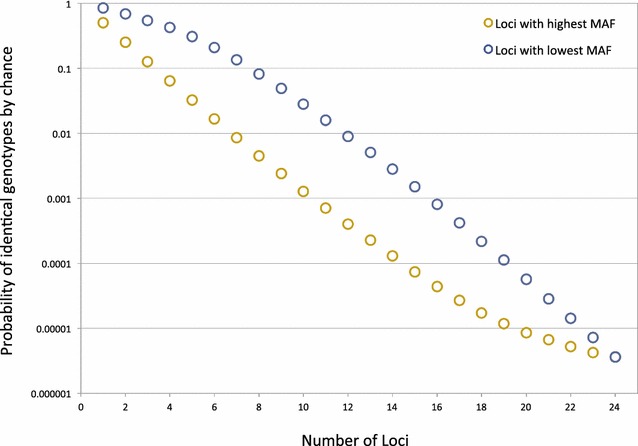
The probability of observing identical multi-locus genotypes by random chance as a function of MAF and the number of loci considered. The probability that two unrelated parasites would have an identical multi-locus genotype by chance (often referred to as identity by state) is shown on the *y-axis* on a logarithmic scale. The probability was calculated using loci with the highest minor allele frequencies (MAFs) (shown in *yellow*), loci with the lowest MAFs (*blue*). See “Methods” section for details on calculation (independence and neutrality assumed)

From the allele frequency data, and assuming independence, we determined the mean number of loci needed for the probability to be <0.01 for observing an identical MLG for unrelated parasite genomes (see Fig. 3). If two parasite genomes were compared at only the loci with the highest observed MAF (and thus the highest resolution), then only seven loci would be needed. Conversely, comparing the parasite genomes at the loci with the lowest MAFs would require typing at five additional loci (12 total) to have the same level of resolution. To be conservative, our analyses subsequently focused on the samples for which 12 or more loci were genotyped (*n* = 94).

### Frequency distribution of multi-locus genotypes: limited evidence of repeat multi-locus genotypes observed between infections

After validating that the loci analyzed could provide a sufficiently high probability of distinguishing unrelated parasite lineages, the frequency of occurrence for the MLGs in the sample set was characterized. The observed MLGs of all samples were compared in all possible pairs, and the samples with the fewest pairwise genetic differences were analyzed further. Notably, there were no instances in which the same MLG was found to be present in more than one individual in our sample. For one individual (individual 1.68.02, see Additional file 2), an identical MLG was observed at consecutive time points. While treatment was offered and provided after a positive RDT result, it is possible that the treatment failed to clear parasites from the bloodstream or was not adhered to by the individual. As a result, we find it more likely that this represents the persistence of a single infection between time points and not a separate infection of this individual with an identical MLG. MLGs were generated at multiple time points for seven other individuals and in each case differed at multiple loci.

Among polygenomic infections, in comparisons between three samples (2.33.04.2, 2.33.08.2, and 1.92.01.2, see Additional file 2), one or more samples were polygenomic but did not exhibit any differences among the loci for which both samples had a single allele. For these cases, it is possible that the MLGs contributing to the polygenomic infection have the same allelic configuration as one present in the other sample. Regardless, such a situation arose in only a small number of comparisons. Due to the lack of definitive instances where independent infections contained identical MLGs, these data strongly indicate that the pattern of repeated clusters of identical MLGs expected from low or sporadic transmission [34] is not present in the Makira region.

### Genetic similarity among polygenomic infections

**Fig. 4:**
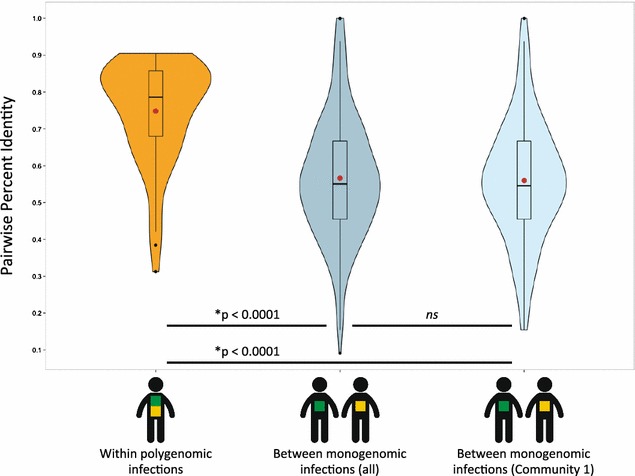
The distribution of genetic similarity among polygenomic and monogenomic infections. *Violin plots* of the distribution of pairwise percent identity among polygenomic and monogenomic infections. Means are marked with a *red point*. *Boxplots* are shown within the violin plots with the median, interquartile range (IQR), and data points more than 1.5 × IQR from the upper or lower quartiles (outliers) marked. *P* values determined by bootstrap sub-sampling (see “Methods” section). *ns* not significant

In order to investigate levels of genetic similarity among the parasite lineages within polygenomic infections, the proportion of loci that differed within polygenomic infections was compared to the average number of differences between randomly selected monogenomic infections. As seen in Fig. 4, a small number of polygenomic infections had significantly fewer shared loci than the average pairwise identity for all monogenomic samples. A possible explanation is that these exceptionally variable polygenomic infections contain more than two parasite MLGs, which would reduce the number of alleles shared by all parasite genomes present in the infection. Despite these few outliers, the distribution of genetic similarity among polygenomic infections was significantly skewed towards higher levels of genetic similarity in comparison to that among monogenomic samples (*p* < 0.0001, bootstrap subsampling to correct for differences in the number of comparisons, see Additional file 3: Figure S1). This result suggests that parasite genomes in polygenomic infections are genetically more closely related than would be expected, and it is consistent with the co-transmission of multiple, related parasites by the bite of a single infectious vector. It is also noteworthy that no significant difference was observed in the average pairwise similarity between all monogenomic samples from community 1 or community 2. Hence this metric suggests an absence of parasite population structure at the community level.

## Discussion

### *Plasmodium falciparum* is prevalent and genetically diverse in the Makira region

Prevalence of *P. falciparum* was 5.7–17.6% at each time point for both sites studied, with 27.8% of individuals and more than 60% of households having at least one positive result over the course of the study. Notably, prevalence in both communities, even at its lowest point (5.7%), was higher than the highest prevalence captured by a recent analysis of national sentinel health sites located in eastern Madagascar (4.9%) [7]. Additionally, the observed prevalence among children under 5 years old seen here (25.4%) was much higher than that found in the national 2013 Malaria Indicator Survey study of 1725 children in eastern Madagascar in the same age group (11.7%) [9]. These observations of prevalence and incidence indicate that transmission frequency, and hence the burden of *P. falciparum*, is much higher at the two sites than would be expected from previous estimates based on regional and national surveillance in Madagascar [1, 2, 7, 35].

Apart from the two study sites, approximately 127,000 people live in the Makira region, all in rural settings with comparable ecological conditions. Additionally, an estimated 86.8% of the 1 million people living in the broader Analanjirofo administrative region (containing the Makira region) also live within similar rural districts [9]. Further, the Analanjirofo region is just one of four such large regions in northeastern Madagascar, which together have a population of over 4.4 million [21]. Accordingly, if the prevalence pattern we observed applies widely across communities/fokontany in the area and the northeastern regions, then this part of Madagascar would be expected to be a significant contributor to the national malaria burden (estimated at a total of 750,000–2,100,000 cases annually [1]). The policy implications are significant; it is possible that government surveillance has focused on coastal and peri-urban sites that are easier to access, or has relied on clinic-based statistics, which may be misleading for remote regions.

An alternative possibility is that the prevalence and incidence seen at the two Makira region sites studied here merely reflects a transient spike. This possibility seems unlikely, because if transmission were sporadic and we simply happened to observe a temporary, unstable peak in transmission in this study, then a pattern of low diversity, frequently repeated MLGs, and fewer polygenomic infections would be expected in the genetic data. In settings of low, unstable transmission, sometimes termed clonal transmission, few MLGs predominate and the reshuffling of those MLGs by recombination or re-assortment is rare. This pattern of clonal transmission has been observed in locations at the periphery of *P. falciparum* transmission such as Panama [35] and in areas of Africa and Asia after interventions have successfully reduced transmission [18, 19].

The pattern observed here is the opposite, as we see very limited evidence of repeated MLGs and a high frequency of polygenomic infections for all subgroups of the sample set (see Table 1). The frequency of polygenomic infections observed in the Makira region was similar, or even higher, than that seen in other high transmission, pre-intervention settings (some examples from studies using SNP-genotyping shown in Table 2).

**Table 2:** Examples of the frequency of polygenomic infections inferred using SNP-genotyping compared across geographic regions and transmission settings

Country	Year	Polygenomic (%)	Transmission setting	References
Madagascar	2013–14	68.1	Present study	Present study
Senegal	2006	41	Pre-intervention	[18]
	2011	26	Post-intervention	[18]
Malawi	2006	76	Pre-intervention	[29]
	2012	68	Post-intervention^a^	[29]
Thailand	2001	63	Pre-intervention^b^	[19]
	2012	14	Post-intervention^b^	[19]

^a^The intervention in Malawi was deemed to be ineffective at reducing transmission

^b^Transmission was estimated to have declined 12-fold after intervention

These indicate that the localities in the Makira region studied here are currently unrecognized foci of high transmission and signal the possibility that other such localities exist in Madagascar. In addition to representing a previously unrecognized source of malaria burden, these localities might serve as sources of reintroduction to areas where interruption of transmission has seen progress. For instance, since the early 1990s the malaria burden has fallen substantially in much of the interior, central plateau of Madagascar [9, 36–39]. Unmonitored hotspots could provide refuges for parasite lineages that could later spillover and compromise these gains. Moreover, recent reports suggest that the burden of malaria has indeed risen broadly since 2011 [9]. This highlights the need for further research and greater surveillance of northeastern Madagascar, and other remote regions currently lacking comprehensive surveillance. Such efforts could determine the extent to which neighboring areas and larger regions also contain foci of elevated prevalence and transmission.

### Potential confounding effects of co-transmission and spatial heterogeneity

Two potential confounding effects that may limit our ability to infer relative levels of transmission from the frequency of polygenomic infections are co-transmission and small-scale spatial heterogeneity in transmission intensity. Co-transmission, where multiple parasite MLGs are taken up by the same mosquito vector and transmitted to the next host, is a mechanism by which polygenomic infections can result from a single transmission event. If co-transmission is common, then the probability of observing polygenomic infections is less dependent on the number of transmission events. Likewise, high spatial heterogeneity in transmission could also cause an increase in the frequency of polygenomic infections without an increase in the number of transmission events by concentrating transmission events in a subset of the population. Hosts within the subset would have a higher probability of being reinfected during the course of an infection and thus more polygenomic infections would be produced.

If these two confounders were present in this study, then it would be expected to see (1) a higher average level of genetic similarity among polygenomic infections than between monogenomic infections and (2) evidence that *P. falciparum* infection was not randomly distributed among the sample population. For co-transmission, serial co-passage of two parasite lineages would provide those lineages with multiple opportunities to recombine with each other. It would be expected that repeated recombination between the parasite genomes would result in progeny with MLGs that were more similar to each other than for parasites that had not recombined as often. Accordingly, if co-transmission were prevalent, it would be expected that the average similarity among the MLGs comprising polygenomic infections would be higher than the average similarity between MLGs chosen randomly from the population. Conversely, if polygenomic infections arose from superinfection (the re-infection of an infected host), then it would be expected that the average similarity among polygenomic infections would be similar to the average between MLGs randomly chosen from the population. Genetic similarity among polygenomic infections was significantly higher than the average between monogenomic infections, consistent with the presence of co-transmission.

As evidence against unusually extreme spatial heterogeneity in our sample, a high percentage (63.0%) of households contained an individual infected at one time point or more. Additionally, there was no evidence that genetic similarity was partitioned between communities. It seems more likely that co-transmission and/or spatial heterogeneity are widespread phenomena in *P. falciparum* populations and therefore their effect is accounted for when comparing populations. Further study of co-transmission and spatial heterogeneity, especially in Madagascar, would serve to better characterize this relationship.

## Conclusion

Despite the abundance of estimates for low to moderate levels of malaria transmission on average regionally and countrywide, we show evidence that the Makira region in northeastern Madagascar is a hotspot of malaria transmission. First, RDT surveys, with RT-PCR confirmation generally validating the results, were performed and an overall level of infection approximately sevenfold higher than the national average and 2 to fivefold higher than existing regional estimates was observed. Next, a panel of SNP markers was validated as variable and informative in Madagascar, and these SNPs were used to estimate the allele and MLG frequencies as well as the frequency of polygenomic infections. A pattern consistent with high, stable transmission was observed. Because few previous studies of *P. falciparum* genetic diversity in Madagascar have been performed, the identification of variable markers, the estimates of minor allele frequencies, and the calculation of the number of loci needed for a given level of resolution will be useful in future studies. Such studies are needed to better define the relative contributions of co-transmission and small-scale spatial heterogeneity to patterns of parasite genetic diversity. Remaining hotspots of *P. falciparum* transmission pose a threat to the recent gains in malaria control in Madagascar, and across Africa, and a better understanding of the genetic correlates of transmission can provide a better method for their identification and monitoring.

## Additional files

**Additional file 1: Table S1.** Contains the minor allele frequency estimates.

**Additional file 2.** Contains the allele calls for the samples analyzed.

**Additional file 3: Figure S1.** Contains the bootstrap re-sample statistics for comparing genetic relatedness.

## Abbreviations

MLG: multi-locus genotype
*Faritra*: the largest administrative regions of Madagascar
RDT: rapid diagnostic test
SNP: single nucleotide polymorphism
RT-PCR: real-time polymerase chain reaction
HRM: high resolution melting
MAF: minor allele frequency
IBS: identity by state
IQR: interquartile range
