# Supplementary material for: Genetic evidence that the Makira region in northeastern Madagascar is a hotspot of malaria transmission
Source: Malar J. 2016 Dec 20;15:596. doi: 10.1186/s12936-016-1644-4 (PMC5175380; doi:10.1186/s12936-016-1644-4)
Supplement: Supplementary file 1 — Additional file 1: Table S1. Contains the minor allele frequency estimates. [file 12936_2016_1644_MOESM1_ESM.docx]

**Supplementary Table 1. Minor allele frequencies (MAFs)**

| **Locus** | **Major Allele** | **Minor Allele** | **MAF** |
| --- | --- | --- | --- |
| A1 | T | G | 0.317 |
| A2 | A | C | 0.444 |
| A3 | C | A | 0.313 |
| A4 | C | G | 0.121 |
| A5 | G | A | 0.369 |
| A6 | G | T | 0.500 |
| A7 | C | T | 0.229 |
| A8 | T | C | 0.279 |
| A9 | T | C | 0.340 |
| A10 | A | C | 0.267 |
| A11 | T | C | 0.393 |
| A12 | T | C | 0.200 |
| B1 | A | G | 0.318 |
| B2 | T | C | 0.417 |
| B3 | C | G | 0.460 |
| B4 | G | A | 0.167 |
| B5 | C | T | 0.403 |
| B6 | A | G | 0.311 |
| B7 | C | A | 0.368 |
| B8 | A | C | 0.463 |
| B9 | A | T | 0.106 |
| B10 | G | A | 0.123 |
| B11 | C | T | 0.457 |
| B12 | G | T | 0.083 |
