# Supplementary figures and images for: Genetic evidence that the Makira region in northeastern Madagascar is a hotspot of malaria transmission
Source: Malar J. 2016 Dec 20;15:596. doi: 10.1186/s12936-016-1644-4 (PMC5175380; doi:10.1186/s12936-016-1644-4)

**S Fig. 1**

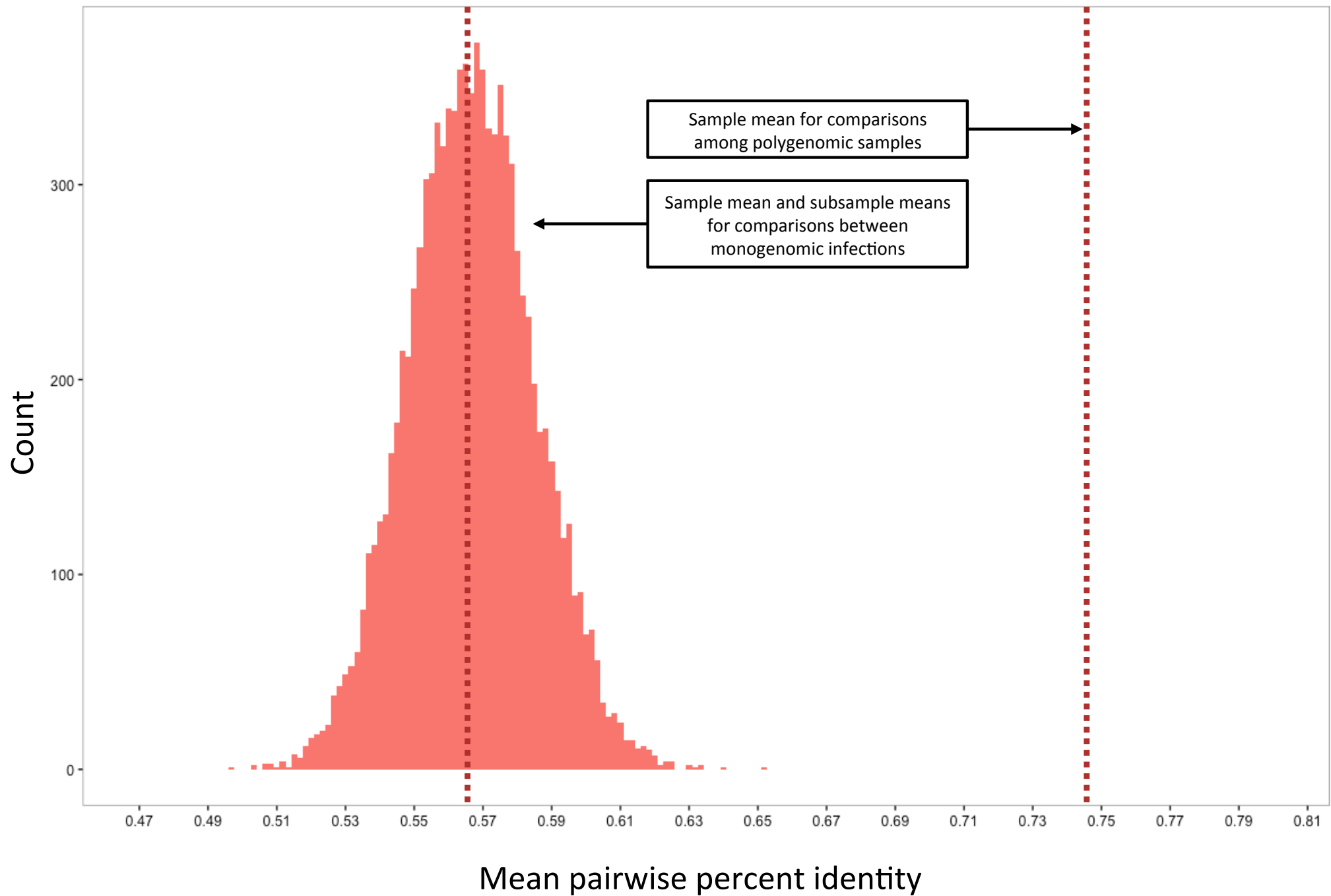

Supplement: Supplementary file 3 — Additional file 3: Figure S1. Contains the bootstrap re-sample statistics for comparing genetic relatedness. [file 12936_2016_1644_MOESM3_ESM.pdf]
